# Editorial: From Single Stem Cells to Organoids, Organ Repair, and Public Health

**DOI:** 10.3389/fcell.2022.849889

**Published:** 2022-02-11

**Authors:** Lon J. Van Winkle, Rebecca J. Ryznar, Philip M. Iannaccone

**Affiliations:** ^1^ Department of Biochemistry, Midwestern University, Downers Grove, IL, United States; ^2^ Department of Medical Humanities, Rocky Vista University, Parker, CO, United States; ^3^ Molecular Biology, Department of Biomedical Sciences, Rocky Vista University, Parker, CO, United States; ^4^ Department of Pediatrics and Pathology, Northwestern University’s Feinberg School of Medicine, Stanley Manne Children’s Research Institute, Lurie Children’s Hospital, Chicago, IL, United States

**Keywords:** drug therapy, organoid, public health, single-cell analysis, stem cells

## Introduction

Teratocarcinomas were shown more than 50 years ago to harbor stem cells that differentiate into components of all three germ layers. Soon, pluripotent cells were obtained from preimplantation embryos and were induced to form three dimensional structures, embryoid bodies recapitulating normal embryonic development. Producing induced pluripotent stem cells (iPSCs) or using adult stem cells, along with directed differentiation is used to develop organized three-dimensional bodies known as organoids that duplicate normal tissues both biochemically and physiologically. When produced from disease state iPSCs, organoids can be used to investigate pathogenesis and therapy. Finally, the regulated heterogeneity of cells as they develop into organoids can now be investigated using single cell RNA sequencing and other emerging genomic technologies. Papers contributed to this Research Topic describe how stem cells and organoids may eventually foster public health.

## Possible Contributions of Organoids to Public Health

In their review, Heremans et al. described work to study tissues comprising the human female reproductive tract using organoids derived from healthy or diseased tissues. Biobanking, gene-editing and multi-omics, tissue and organ regeneration, interactions with pathogens, drug testing, and precision therapy all can be evaluated using these models. The authors describe models and conditions needed to maintain healthy tissues or alternatively produce disease states, such as endometriosis and endometrial cancer.

Zhang et al. consider the ways in which intestinal organoid (IO) technology provides new opportunities to study intestinal cell differentiation, interactions, organogenesis, and function. Moreover, IOs are used to study inflammatory bowel disease, colorectal cancers and other malignancies, gastrointestinal infections, and genetic diseases including cystic fibrosis. These authors also describe current shortcomings of organoid research using bibliometric analyses, including the absence of other pertinent structures, such as the vascular system and associated cells, and they recommend that co-culture systems be developed. The organoids block external effectors, such as drugs, from reaching the apical side of the IOs, an important future research direction.

In their mini review, Sun et al. discuss skin complexity and components, such as hair follicles and sweat glands, that are missing from most efforts to generate functional human skin *in vitro*. Human skin production *in vitro* is an important source of tissues for therapy and study. Of particular interest is regeneration of eccrine sweat glands in human skin produced *in vitro* (and as discussed by Lin et al. in their review). Multiple signaling pathways, that govern cellular differentiation into eccrine sweat gland cells, are discussed. Sun et al. discuss the sources of skin cells and skin organoids, the microenvironment for these cells and the challenges and opportunities involved in producing skin organoids that are multifunctional.

In their review, Fernandes et al. describe the use of human and nonhuman iPSCs to generate mini-brain organoids to probe the complexity of human-specific brain development and human brain evolution. Organoids can be generated representing different brain regions, allowing the study of interactions between combinations in culture. A multiplicity of genes important to brain development are differentially expressed spatiotemporally in humans vs. nonhuman primates. The transcriptome can be manipulated and perturbed in brain organoids using gene editing technologies. These studies will reveal not only how mechanisms of brain development differ among species and unique mechanisms driving brain disorder pathophysiology, but also processes, such as neoteny, that have been employed during human brain evolution.

In their research article, Erni et al. used cochlear organoid cultures and other means, such as explants of the organ of Corti, to show that two novel gamma-secretase inhibitors, termed CPD3 and CPD8, increased cochlear hair cell numbers. Their preclinical data support the notion that CPD3 might be used to restore hearing in clinical trials. Since hair cell degeneration is a major contributor to hearing loss among five percent of people in the world, successful therapeutic development would substantially improve the health of affected individuals and public health in general.

## Use of Stem Cells Directly to Promote Human Health

Three research articles report successful efforts to produce and utilize stem cells clinically. First, Chakritbudsabong et al., showed that exogenous LIN28 supports porcine iPSC pluripotency and proliferation. Successful porcine iPSC production has lagged that of other species and prevented establishment of this model of human development and differentiation. Organs derived from porcine iPSCs might be used eventually to help to produce organs for human transplant. Moreover, porcine iPSCs will help improve veterinary medicine therapies and agricultural production.

For humans, Rabesandratana et al. demonstrated that hiPSCs can be used to generate retinal ganglion cells (RGCs). This use of hiPSCs is important because RGCs themselves are not easily located and grown *in vitro*. RGCs produced from hiPSCs survived for nearly a month when the authors injected them into the vitreous of mice experiencing optic neuropathy. Thus, these RGCs might eventually be used to promote human health by preventing or even reversing blindness.

Lan et al. describe how exosomes produced by bone marrow stem cells (BMSCs) foster their own osteogenic differentiation on titanium surfaces. This differentiation is essential to the successful production of dental implants and is impeded by medical conditions like diabetes, osteoporosis, and others. Loss of teeth from deterioration or accident leads to malocclusion, difficulty eating, and significant bone loss in the jaw. Ameliorating these effects will improve the health of millions of people with obvious impacts on the public health.

Three review papers also describe the possible use of stem cells directly to promote human health. To treat type 1 diabetes mellitus, beta-cell function can be restored through islet transplantation. The supply of beta-cells is, of course, limited by donor availability. However, Szlachcic et al. consider how an inexhaustible supply of beta-cells might be produced from hiPSCs. These authors consider how single-cell RNA sequencing (scRNA-Seq) could be used better to understand normal pancreas development and, specifically, beta-cell differentiation. This understanding might then be used to produce better sources of beta-cells for individuals from whom the hiPSCs were derived. The consequences of such technology for worldwide public health could be enormous, since the costs of diabetes, as measured both monetarily and regarding human suffering, are extremely large. In 2020, the cost to care for persons in the US with type 1 diabetes was projected to be $203 billion over a ten-year period (Sussman, M., Benner, J., Haller, M. J., Rewers, M., and Griffiths, R. (2020). Estimated lifetime economic burden of type 1 diabetes. *Diabetes technology and therapeutics* 22, 121–130).

Similarly, mesenchymal stem cells (MSCs) transplantation could be used to treat premature ovarian failure (POF), as described in the review by Wang et al. The incidence of POF has increased to about ten percent of women in recent years apparently owing, in part, to greater life stresses. MSC transplantation in animals has restored ovarian function, apparently through their differentiation into oocytes, and makes us optimistic that fertility can be restored in this way in women with POF. MSCs have already been used clinical to treat many human diseases including blood, skin, and cardiovascular disorders.

Liver injury, regeneration, and diseases, including cancer, might also be fostered/treatable by properly activating stem cells. Several types of cells are involved in liver regeneration, and their balanced regulation is essential to this process. For example, c-kit positive hepatocytes, oval cells, and bile epithelial cells promote liver repair by differentiating into different types of cells depending on the nature of the injury. But c-kit positive mast cells are associated with primary sclerosing cholangitis, and c-kit overexpression may result in malignancy. Hence, Wang et al. concluded that c-kit is “a double-edged sword in liver regeneration and diseases.”

